# DLX1008 (brolucizumab), a single-chain anti-VEGF-A antibody fragment with low picomolar affinity, leads to tumor involution in an *in vivo* model of Kaposi Sarcoma

**DOI:** 10.1371/journal.pone.0233116

**Published:** 2020-05-14

**Authors:** Anthony B. Eason, Sang-Hoon Sin, Mohsin Shah, Hong Yuan, Douglas J. Phillips, Miriam Droste, Abdijapar Shamshiev, Dirk P. Dittmer

**Affiliations:** 1 Lineberger Comprehensive Cancer Center, The University of North Carolina at Chapel Hill School of Medicine, Chapel Hill, North Carolina, United States of America; 2 Department of Microbiology and Immunology, The University of North Carolina at Chapel Hill School of Medicine, Chapel Hill, North Carolina, United States of America; 3 Biomedical Research Imaging Center, The University of North Carolina at Chapel Hill School of Medicine, Chapel Hill, North Carolina, United States of America; 4 Cell Medica, Zurich, Switzerland; Loyola University Health System, UNITED STATES

## Abstract

Kaposi Sarcoma (KS) is among the most angiogenic cancers in humans and an AIDS-defining condition. KS-associated herpesvirus (KSHV) is necessary for KS development, as is vascular endothelial growth factor (VEGF-A). DLX1008 is a novel anti-VEGF-A antibody single-chain variable fragment (scFv) with low picomolar affinity for VEGF-A. *In vivo* imaging techniques were used to establish the efficacy of DLX1008 and to establish the mechanism of action; this included non-invasive imaging by ultrasound and optical fluorescence, verified by post-mortem histochemistry. The results showed that DLX1008 was efficacious in a KS mouse model. The NSG mouse xenografts suffered massive internal necrosis or involution, consistent with a lack of blood supply. We found that imaging by ultrasound was superior to external caliper measurements in the validation of the angiogenesis inhibitor DLX1008. Further development of DLX1008 against VEGF-dependent sarcomas is warranted.

## Introduction

Kaposi Sarcoma (KS) is among the most angiogenic cancers in humans. Kaposi Sarcoma-associated herpesvirus (KSHV, or human herpesvirus 8) is the causative agent of KS and a driver of KS angiogenesis (reviewed in [1]). KSHV infection of primary human endothelial cells [2] in culture induces expression of Vascular Endothelial Growth Factor A (VEGF-A) protein, as well as three main types of VEGF receptors: VEGF-R1/FLT-1, VEGF-R2/FLK-1/KDR, and VEGF-R3/Flt-4 [3, 4]. This establishes a positive feedback loop essential for tumor growth (reviewed in [5]). VEGF-A, found at vastly elevated levels in KS biopsies, is thought to be responsible for the angiogenic phenotype of KS and KSHV-infected endothelial cells [6]; VEGF-R2/FLK-1/KDR is also expressed in KS lesions [7]. KS appears to be more dependent on VEGF-A than other cancers [8–10], and KSHV reprograms endothelial cells to increase their responsiveness to VEGF-A. Thus, KS is a relevant cancer in which to study the mechanism of action and efficacy of angiogenesis inhibitors. KS is phenotypically intermediate between reactive polyclonal hyperplasia and fully transformed monoclonal neoplasia. KSHV itself does not transform primary or life-span-extended human cells in culture. KSHV confers only a subtle survival advantage to human cells [11–13], though the virus fully transform rodent cells [14, 15]. The biology of the virus fits the pathology of the cancer. Higher-grade KS is associated with significant morbidity and mortality, while “classic” KS of older Mediterranean men is often indolent [16, 17]. Some KS lesions respond to single agent VEGF inhibitors such as bevacizumab, a recombinant monoclonal antibody (mAb) directed against VEGF-A [18–20]; other lesions require aggressive cytotoxic chemotherapy. The same dichotomy applies to B cell malignancies caused by KSHV. This prompted us to explore whether novel VEGF-neutralizing biologics would have pre-clinical activity in KS models.

Single chain variable antibody fragments (scFvs) are among the smallest possible antibody fragments which retain the entire antigen binding site. However, they are typically too unstable to use as therapeutics [21]. The PENTRA^®^Body scFv platform overcomes these limitations, allowing the creation of humanized scFvs with high stability. DLX1008, a VEGF-binding PENTRA^®^Body, is the same molecule as brolucizumab (Novartis), which has shown promising results in phase 3 clinical trials for age-related macular degeneration [22]. Two principal reasons motivated the study of DLX1008 in KS. First, DLX1008 has 30-fold higher affinity for VEGF-A-165 as compared to bevacizumab (1 pM, compared to 32 pM). It has equivalent or superior inhibition of receptor dimerization and binding when compared to bevacizumab [23]. Second, DLX1008 is smaller (26 kDa) compared to bevacizumab (150 kDa). It has a different pharmacokinetic profile and biodistribution. Brolucizumab, the same molecule as DLX1008, has a serum half-life of 5.6 ± 1.5 hours in non-human primates [24]. The serum half-life in non-human primates is provided for comparison. It was not indicative of the half-life in mouse models, as there are species-specific differences which may affect clearance. Hence, we repeated the half-life studies here. Biodistribution is governed primarily by molecular weight [25, 26]. The extracellular matrix has a large resistance to full-length antibody diffusion, causing antibodies to preferentially follow the flow of fluid through convective extravasation. A low rate of passive diffusion limits their distribution and might preclude mAbs from reaching tumors that are under-vascularized or where the vasculature is disordered [27]. This is due to the relatively smaller size of pores in the capillary through which they extravasate and return compared to the larger pores in the lymphatic vessels; the high-pressure gradient created forces fluid into low-pressure areas around the lymphatic vessel so that mAbs leave the tissue faster than they entered it. Both of these factors result in a low concentration of mAbs in the interstitial space relative to blood and/or lymphatic levels (reviewed in [28–30]). Compared to normal organ vasculature, tumor microvessels are immature, display insufficient pericyte stabilization, and are overly permeable. Consequently, exogenously administered mAb has been shown to localize in tumor tissue close to the supplying vessels, while fragments, such as scFvs, penetrate the tumor homogenously [31]. Lack of tumor penetration has been proposed as one reason for the limited and highly variable efficacy of bevacizumab in KS. Even when directly injected into upper-respiratory KS lesions, bevacizumab, though well-tolerated, had no effect on tumor size compared to the control group [32]. This is demonstrative of the limitations of whole-antibody anti-VEGF therapy on vascular tumors and motivated our exploration of antibody fragments.

At present, no permanent cell lines derived directly from KS lesions exist. Patient-derived explants (PDX) of KS, likewise, do not exist. This hurdle has made it difficult to study KS angiogenesis. Current experimental designs provide important insights. The predominant experimental models are based on PEL cell lines or on cell lines that have been infected with KSHV in culture. These cells lines represent multiple lineages and stem from primate and rodent species, including B cells, endothelial cells, epithelial cells. Each model recapitulates an important aspect of KS and KSHV biology [2, 4, 14, 15, 33–40]. A second hurdle in studying KS is that angioproliferative KS lesions typically consist of a heterogeneous mélange of fibroblasts, smooth muscle cells, infiltrating inflammatory leukocytes, and endothelial cells (ECs); any one or all pose targets for anti-tumor therapy. Perplexingly, although KSHV is consistently and exclusively found in ECs of spindle morphology, neighboring and morphologically indistinguishable ECs are virus-free. Thus, approaches that target virus-negative as well as virus-positive cells in the lesion may show clinical benefit.

To explore the efficacy of DLX1008, we tested a population of cells *in vivo* which we developed as a derivative of a KSHV-infected, transformed cell population of mixed origin [41]: mSLK-KSHV. These cells represent a fusion of KSHV-infected, telomerase-immortalized vein endothelial cells [34] with the SLK/Caki-1 cell line. The mSLK-KSHV cells rapidly form tumors in mice and are also unusually permissive for KSHV infection [42–44]. *In vivo*, they maintain the KSHV genome in the absence of selection. The mSLK-KSHV cells maintain EC markers found in primary KS lesions, as well as many molecular signaling pathways characteristic of KS [41, 45]. They maintain the same VEGF feedback loop and dependency as observed in clinical KS and are predictive of clinical drug efficacy, e.g. for the mTOR inhibitor rapamycin [46]. We hypothesized that utilizing this mouse model to test the efficacy of DLX1008 would result in an inhibition of tumor growth, which could be further elucidated using *in* vivo imaging technology. Combining the model with detailed imaging studies showed that DLX1008 inhibited blood flow to the tumor, resulting in involution and tumor necrosis from the inside out. Tumor penetrance may have been aided by the extremely small size of this antibody fragment. Further clinical studies of DLX1008 in KS seem warranted.

## Materials and methods

### Key resources

Details regarding the reagents and resources utilized in described experiments can be found in Table 1.

**Table 1:** Key resources table.

REAGENT or RESOURCE	SOURCE	IDENTIFIER
**Antibodies**		
Rabbit monoclonal anti-VEGFR-2	Cell signaling	Clone 55B11
DLX1008 anti-VEGF-A scFv	Provided by Cell Medica	N/A
Mock scFv	Provided by Cell Medica	DLX1084
Bevacizumab	Roche	Avastin
**Chemicals, Peptides, and Recombinant Proteins**		
Matrigel, Growth factor reduced (GFR)	Corning	354230
Protocol 10% Neutral Buffered Formalin	Fisher Scientific	032–059
EnVision Flex Wash Buffer	Agilent	K800721-2
Histochoice Clearing Agent	VWR	VWRVH103-1L
**Critical Commercial Assays**		
MycoAlert Plus Mycoplasma Detection kit	Lonza	LT07-701
IntegriSense 750 Fluorescent Imaging Agent	PerkinElmer	NEV10873
IRDye 800CW RGD Optical Probe	LI-COR	926–09889
VectaStain Elite ABC-HRP Kit (Rabbit IgG)	Vector Laboratories	PK-6101
Avidin/Biotin Blocking Kit	Vector Laboratories	SP-2001
ImmPACT NovaRED Peroxidase (HRP) Substrate	Vector Laboratories	SK-4805
Affymetrix Genome-Wide Human SNP 6.0 Array	Affymetrix	GPL6801
**Experimental Models: Cell Lines**		
Human: mSLK-KSHV	This paper based on [41]	N/A
**Experimental Models: Organisms/Strains**		
Mouse: NSG: NOD.Cg-Prkdc^scid^ Il2rg^tm1Wjl^/SzJ	The Jackson Laboratory	005557
Mouse: C57BL/6J	Janvier Labs	C57BL/6JRj
Mouse: Athymic nude: A/A Tyr^c^/Tyr^c^ Foxn1^nu^/Foxn1^nu^	The Jackson Laboratory	002019
**Software and Algorithms**		
SoftMax Pro	Molecular Devices	v5.4
Living Image	Caliper LifeSciences	v4.3.1
Zen 2 Core	Carl Zeiss AG	v2
ImageJ	[47]	v1.51
R	[48]	v3.5.2
R package ‘survival’	[49, 50]	v2.38
R package ‘survminer’	[51]	v0.4.3
R package ‘dplyr’	[52]	v0.7.8
Partek Genomics Suite	Partek Incorporated	v7.18.0723

### Experimental model details

#### Cell culture model

mSLK-KSHV cells were grown in Dulbecco’s Modified Eagle Medium (DMEM) supplemented with 10% v/v fetal bovine serum (FBS), 100 U/mL penicillin, 100 μg/mL streptomycin, and 2 mM L-glutamine. The tumor cells were adapted to growth in NOD.Cg-Prkdc^scid^ Il2rg^tm1Wjl^/SzJ (NSG) mice by unilateral subcutaneous flank injection of 1 x 10^5^ thrice-washed cells in 200 μL of phosphate buffered saline (PBS). Upon palpable tumor formation, the tumor was removed in a sterile biosafety cabinet, dissected from connective tissue, and minced in DMEM growth medium supplemented with 10% FBS and 100 U/mL nystatin. After 3 days, medium was replaced with complete DMEM growth medium and split into a new flask. All cells were verified to be negative for mycoplasma using MycoAlert Plus Mycoplasma Detection Kit (Lonza). Cells were grown at 37°C and 5.0% CO_2_ in humidified incubators.

#### Tumor studies

Experiments were performed using NSG mice, which were obtained from the Jackson Laboratory and maintained by the UNC Animal Studies Core (ASC) in UNC Division of Comparative Medicine (DCM)-monitored space. Mice used in the study were group-housed in aseptic conditions and monitored daily for changes in health or behavior. Males and females were housed separately in groups of five and given ad libitum access to fluorescence-free sterilized mouse chow and water. Tumors were seeded via unilateral subcutaneous flank injections of thrice-washed mSLK-KSHV cells suspended in 1:1 sterile PBS and growth-factor reduced (GFR) Matrigel (Corning Cat# 354230).

Comparative genomic hybridization (CGH) was performed using the Affymetrix CGH v6.0 (Geo profiles Platform GPL6801) following the manufacturer’s instructions to establish the unique genotype of mSLK-KSHV. Additional data were from GSE32264 [53]. Analysis was conducted using Partek Genomics Suite v7.18.0723.

### Method details

#### DLX1008 half-life determination

Experiments were performed using adult male C57BL/6J mice, which were obtained from Janvier Labs (Le Genest St Isle, France) and maintained in a temperature-controlled room (20–24°C), with a 12h light/12h dark cycle. Mice were given *ad libitum* access to maintenance diet (ssniff^®^ R/M-H, 10 mm) and water. Sixteen mice were given a single intravenous administration of 10 mg/kg DLX1008, formulated in PBS, pH 7.2, at 2 mg/mL. At each timepoint, a volume of 120 μL of whole blood was collected into lithium heparin-containing tubes from the retrobulbar venous plexus under short isoflurane anesthesia. Whole blood samples were stored on ice before centrifugation (10 minutes at 3000 x G, 4°C). Plasma samples were frozen and stored at -20°C before analysis. Blood was collected at two timepoints for each mouse, either 5 minutes and 2 hours, 15 minutes and 4 hours, 30 minutes and 8 hours, or 1 and 24 hours after DLX1008 administration. Four mice were sampled per timepoint. DLX1008 concentration in plasma samples was determined by ELISA. Maxisorp plates (ThermoFisher Scientific) were coated overnight at 4°C with 2μg/mL of a mouse monoclonal anti-DLX1008 antibody. Wells were blocked for 1.5 hours at room temperature with PBS containing 0.05% Tween-20 (PBS-T). Mouse plasma was diluted in PBS-T and incubated in wells for 1 hour. In order to construct a standard curve, DLX1008 was diluted in PBS-T to between 0.38–12 ng/mL, and the standard dilutions were incubated in wells for 1 hour. DLX1008 was detected using a biotinylated mouse monoclonal anti-DLX1008 antibody which binds to a different epitope of DLX1008 than the coated antibody, followed by streptavidin-HRP and TMB substrate solution. Unknowns were calculated from the standard curve using a 4-parameter fit in SoftMax Pro 5.4 (Molecular Devices). The lower limit of quantification for the standard curve was calculated as the lowest standard concentration which fulfills the following criteria: after blank subtraction absorbance is a positive value, coefficient of variation (CV) of measured replicates is in the range of ± 30%, average of back calculated standards is within ± 30% of the expected value.

#### Tumor implantation and treatment

mSLK-KSHV cells were harvested from culture during logarithmic growth and washed three times in PBS. Viability and concentration were determined by trypan blue visualization. The cells were suspended in PBS at a concentration of 5 x 10^6^ cells per mL, then chilled to 4°C and gently mixed at a 1:1 ratio with GFR Matrigel (Corning) to a final concentration of 2.5 x 10^6^ cells per mL. NSG mice were subcutaneously injected unilaterally on the flank with 200 μL of cell suspension (5 x 10^5^ cells), and the tumor was allowed to establish for 3 days prior to starting treatment. The location of the tumors was chosen to minimize interference with the animals’ normal behavior, ambulation, and life patterns. Mice were assigned into three groups consisting of 10 females (trial 1) or 5 males/5 females (trial 2). The animals were injected 5 days per week (M/T/W/Th/F) intraperitoneally with either 15 mg/kg/day of DLX1008, 15 mg/kg/day of isotype control scFv, or PBS. The treatment was 21 days in duration for trial 1, and it was extended to 41 days in duration for trial 2. During the treatment, animals were monitored daily for tumor size, weight gain, malaise, and motility issues. Animals were classified and approved by IACUC under USDA Pain and Distress Category C: Slight or momentary pain or distress or no pain or distress. In addition, we followed the Body Condition Scoring system as outlined in the UNC IACUC Standard Operating Policies and Guidelines and observed the mice for difficult and/or rapid breathing, jaundice, anemia (pale extremities) and swollen abdomen via palpation and visual examination. A body condition score indicating unhealthy weight loss, indications of pain or distress greater than that in USDA Category C, or a tumor size of 2 cm in any dimension was indicative of the individual humane endpoint if reached prior to the end of treatment. Therefore, upon malaise, body score equal or < 2, or the development of macroscopic tumors (2 cm in any dimension) mice were euthanized via a controlled-flow carbon dioxide chamber followed by approved secondary cervical dislocation. Tumor size was measured daily via calipers and recorded as volume = (length*width^2^)/2. Mice were euthanized and tumors/kidneys were collected upon treatment cessation or upon reaching the humane endpoints described above in trial 1; mice were permitted to reach humane endpoints after treatment cessation in trial 2, at which point mice were euthanized and tumors/kidneys were collected. Mice were observed after treatment cessation in trial 2 in order to ascertain longitudinal treatment effects on time required to reach the endpoint. Two weeks after treatment, all remaining animals were considered to have reached endpoint (54 days maximum from treatment start). All mice meeting humane endpoint criteria were euthanized within 1–2 hours of the determination; no animals died prior to meeting endpoint criteria. 60 adult mice were used in total, and all were euthanized as described.

#### Ultrasound imaging

Ultrasound images of tumors were collected during trial 2, at two time points; treatment days 7–9 were considered the initial time point, and days 41–43 were considered the final time point. Mice were imaged over the course of three days with two cages (up to 5 mice per cage) imaged per day in a randomly assigned cage order. A high-resolution preclinical ultrasound imaging system (Vevo 2100, FUJIFILM VisualSonics, Inc) was utilized to conduct ultrasound imaging on tumor-bearing mice. Mice were anesthetized with isoflurane (1.5%)/oxygen inhalation during imaging. The tumor area was briefly chemically depilated, rinsed, and covered with ultrasound imaging gel. A MS-550D ultrasound transducer with center frequency of 40 MHz and 14 mm tissue penetration was placed on the tumor region. B-mode imaging was taken to capture the tumor cross-section in the middle along the tumor long axis; to do this, ultrasound was used to determine the longest axis of the tumor and the exact length. The probe was then placed with ultrasound gel at the halfway point of maximum tumor length, in a view perpendicular to the length axis. Utilizing the instrument B-Mode setting at maximum image depth, the entire tumor cross-section as indicated by visible tumor capsule boundaries was included in the image. Gain was set to 22 dB, and sensitivity and persistence were set to “high.” Individual images were analyzed with ImageJ by creating a consistent region of interest (ROI) within the tumor capsule and centered below the skin. We then applied thresholding to the pixel intensity, such that solid tissue (white on ultrasound) was highlighted in green and was excluded from quantification. Non-solid/necrotic tissue (black on ultrasound) was quantified as a percentage of all pixels and translated as an approximation of percentage necrosis.

#### Optical imaging

Optical fluorescent images of integrin expression in the tumor-bearing animals were collected at the endpoint of trial 2, over the course of 4 days following treatment cessation. Animals were imaged utilizing an *in vivo* optical imaging system (IVIS Lumina II, PerkinElmer, Inc) with IntegriSense 750 near-infrared (NIR) fluorescent integrin probe (PerkinElmer, Inc). A 100 μL bolus of IntegriSense 750 solution (2 nmol/100 μL in PBS) was administered via lateral tail vein injection under isoflurane anesthesia. Fluorescence imaging was taken before injection to determine the background, immediately after injection to verify the intravenous injection, and at 24 hours post-injection to quantify the integrin expression. Mice with unsuccessful or perivascular injections, indicated by a lack of systemic fluorescence above background levels, were removed from the optical imaging data collection. Images were analyzed using Living Image v.4.3.1 (PerkinElmer, Inc) in units of radiant efficiency, and regions of interest (ROI) were automatically created to be inclusive of values with at least 21% of peak pixel intensity. Average radiant efficiency of the ROI was calculated to represent integrin expression.

#### Immunohistochemistry

Tissues were fixed in 10% neutral buffered formalin (NBF) at 4°C for 72 hours. Tissues were then processed, embedded in paraffin wax, cut to 4-μm thickness, mounted onto glass slides, and stained for H&E by the UNC Animal Histopathology and Laboratory Medicine Core. Prior to staining, slides were dried at 58°C for 3 hours. Using the Dako PT Link instrument (Agilent, Santa Clara, CA), slides were pretreated at 75°C in 10 mM sodium citrate buffer adjusted to pH 6.0 with 0.05% Tween-20. The buffer temperature was raised to 96°C and held at that temperature for 25 minutes in order to perform antigen retrieval, and then the temperature was lowered back to 75°C. Slides were agitated in 1X EnVision Flex Wash Buffer (Agilent) at room temperature to remove excess paraffin; slides were then incubated in the wash buffer for 5 minutes. Slides were washed in PBS for 5 minutes, followed by a 10-minute incubation in 3% hydrogen peroxide to quench endogenous peroxidase. PBS washes were performed between all the following steps, and incubations were performed in a humidified chamber. Samples were incubated for 30 minutes with 1.5% goat serum from VectaStain Elite ABC Kit (Vector Laboratories, CA, USA) diluted in PBS supplemented with 1% bovine serum albumin (BSA), 0.1% cold water fish skin gelatin, 0.1% Triton X-100, 0.05% Tween-20, and 10% avidin from Avidin/Biotin Blocking Kit (Vector Laboratories). Sections were incubated at 4°C overnight in either control diluent, consisting of PBS with 10% biotin, 1% BSA, 0.1% cold water fish skin gelatin, and 0.1% Triton X-100, or the primary antibody diluted in the same solution. Primary antibody dilution was 1:300 for VEGFR-2 (Table 1). Sections were then incubated with VectaStain Elite ABC Kit-provided biotinylated goat anti-rabbit secondary antibody, prepared according to the manufacturer’s instructions, for 30 minutes. Kit-provided ABC (avidin-biotin complex) amplification reagent was prepared according to manufacturer instruction and applied to sections for 30 minutes. Slides were developed with ImmPACT NovaRED Horseradish Peroxidase (HRP) Substrate (Vector Laboratories) for 5 minutes and quenched with nanopure water. Mayer’s hematoxylin was used as a counterstain by submerging slides for 45 seconds, followed by a brief agitation in 1% ammonium hydroxide solution and rinsed with nanopure water. Slides were then dehydrated and cleared on a Leica ST4020 Linear Stainer instrument (Leica Biosystems, Nussloch, Germany) following a protocol of 250 seconds each in 50%, 70%, 80%, 95%, and 100% ethanol followed by four changes of Histochoice clearing agent (VWR International, PA, USA). Slides were mounted with a glass coverslip using hard-set mounting medium and allowed to cure overnight prior to imaging.

#### Microscopic analysis

Slide images were acquired using bright field microscopy using a Leica DM4000B upright microscope (Leica Microsystems, Wetzlar, Germany) fitted with either a HC PL apochromatic 10X objective with 0.40 numerical aperture (NA), HC PLAN apochromatic 20X objective with 0.70 NA, or HCX PL apochromatic 40X objective with 0.85 NA. Image capture was performed using an Axiocam 105 Color 5-megapixel CMOS digital microscope camera (Carl Zeiss, Jena, Germany) with a 0.55X magnification c-mount attachment. A PC with Windows 10 Enterprise operating system was used to run Zen 2 Core software (Carl Zeiss) for image capture. Slides stained for VEGFR-2 were visually compared with positive control clinical KS references and no-primary controls to qualitatively verify expression of the receptor. Cell culture images were acquired using bright field microscopy with phase contrast, using a Leica DMIL inverted microscope (Leica Microsystems, Wetzlar, Germany) fitted with a HI PLAN achromatic 10X objective with 0.25 NA. Stand-alone image capture was performed using an Axiocam ERc 5s Color 5-megapixel CMOS digital microscope camera (Carl Zeiss, Jena, Germany) with a 0.55X magnification c-mount attachment.

#### Quantification and statistical analysis

For tumor studies, cage randomization was performed by assigning 6 mouse cages consecutive integers between 1 and 6 and utilizing a random number generator to determine the order in which cages would be selected for imaging over the course of 3 days. Kaplan-Meier (KM) survival analysis was performed using a log-rank statistical analysis with R version 3.5.2 and the R packages ‘survival,’ ‘survminer,’ and ‘dplyr’ [48–52]. Comparison of the ultrasound necrosis percentages was done using multiple comparison of means with Dunnett contrasts. For PK analysis, we assumed a two-phase half-life with estimation by biexponential model using the R package ‘pk’.

#### Ethical approval

Human control KS biopsy tissues were obtained as anonymized specimens from a biorepository. Use of deidentified biorepository specimens was classified as non-human subject research by UNC IRB and was not subject to review. All applicable international, national, and institutional guidelines for the care and use of animals were followed. All procedures performed in studies involving animals were in accordance with the ethical standards of UNC Institutional Animal Care and Use Committee (IACUC) or the Landesamt für Gesundheit und Verbraucherschutz, Abteiling Lebensmittel- und Veterinärwesen, Saarbrücken, Germany. This study was approved under UNC IACUC protocol number 16–040.0. Animals experiencing unrelieved pain or distress prior to the endpoint, as defined by institutional policy, must be humanely euthanized, unless an exception to policy is requested and approved. No exception to this policy was requested or granted. Personnel interacting with mice were registered as animal handlers and certified in mouse handling and techniques by UNC Office of Care and Animal Use in compliance with U.S. federal law.

## Results

### Validation of VEGF-1 dependence in a KS tumor model

At present, there exists no definitive angiogenesis model for KS, as no patient derived cell lines exist, and KSHV does not transform primary human cells. We explored a mouse-adapted cell line, mSLK-KSHV, which reflects a fusion of KSHV-infected endothelial cells [34] and a derivative of the renal-cell carcinoma cell line Caki-1 [44]. The mSLK-KSHV line combines desirable properties of each parental line: (i) it is KSHV positive, (ii) expresses EC markers, and (iii) it forms reproducible, highly angiogenic tumors in immunodeficient mice [54].

To document genomic alterations, whole genome copy-number analysis was performed (S1 Fig). tert-immortalized HUVEC cells (TERT HUVEC) showed no chromosome abnormalities. KSHV TERT HUVEC, showed a limited number of abnormalities. By contrast, TIVE-E1/L1 cells, and their mouse tumor-derived explant mSLK-KSHV, showed dramatic chromosomal abnormalities. Chromosomes 5, 14, 18, 20, and 22 show a pattern of chromosomal abnormalities also seen in Caki-1 and SLK cells. This result is consistent with limited STR typing, which Caki-1 as the origin. Other chromosomes, however, did not match the Caki-1 chromosomal pattern or were unchanged (chr. 15, 21). Overall, the mSLK-KSHV is the result of selection for subcutaneous growth in immunodeficient mice, clonal expansion after explantation from the murine graft, and single cell screening for high KSHV LANA protein expression. It maintains key characteristics of KS tumor cells, such as dependence on VEGF-A and expression of αVβ3 integrin. Therefore, mSLK-KSHV represent a suitable pre-clinical model to evaluate anti-KS modalities *in vivo*.

**Fig 1:**
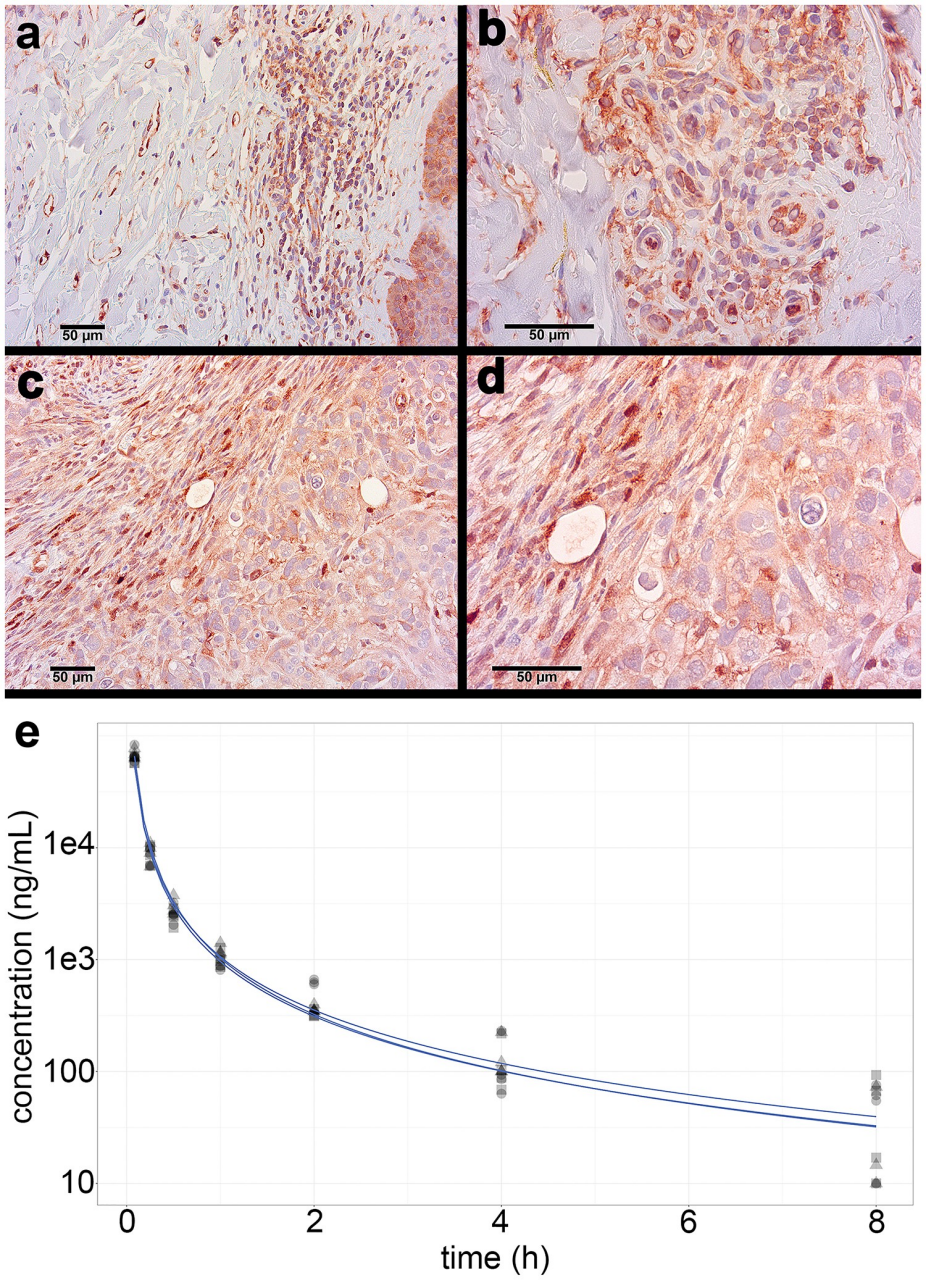
VEGFR-2 is highly expressed in mSLK-KSHV xenograft tumors, and DLX1008 has a short half-life *in vivo*. Human KS biopsy tissue positive controls, immunostained for VEGFR-2, contain spindle-cell infiltrates with focal red staining of the tumor cells, shown at low magnification (A) and a different biopsy at high magnification (B). Treated (DLX1008) and untreated (isotype control, PBS) xenograft tumors of mSLK-KSHV in NSG mice were immunostained red for VEGFR-2, and a representative tumor section from the isotype control-treated group is shown at low (C) and high (D) magnification. Counterstain in blue, all scale bars 50 μm. Average serum concentration of DLX1008 over time is modeled through a biphasic analysis (E) from preliminary mouse data after IV injection. Data is shown from three independent experiments.

To confirm that mSLK-KSHV binds VEGF-A *in vivo*, we performed immunohistochemistry. Tumors were compared to KS biopsy reference tissues, which demonstrated specific VEGFR-2 staining localized to the KS spindle cells (Fig 1A and 1B). There was similarly robust VEGFR-2 staining in mSLK-KSHV tumors (Fig 1C and 1D). Differences in morphology were likely due to the heterogeneous environment of KS lesions compared to the homogenous environment of purified single cells embedded in Matrigel. All groups of mice expressed VEGFR-2 within the tumor capsule, in all viable cells within the implant, and at levels similar to that observed in untreated human cutaneous KS.

### DLX1008 slows tumor growth and decreases tumor health *in vivo*

To verify that DLX1008 was delivered and circulated in the mice, we conducted a PK analysis. A bolus of 10 mg/kg DLX1008 was injected IV into 16 mice and blood collected longitudinally at 0.083, 0.25, 0.5, 1, 2, 4, and 8 hours, for 4 mice per timepoint. Plasma concentration of DLX1008 was determined by ELISA. (Fig 1E) shows DLX1008 concentration for each of the three independent experiments. These experiments established a half-life of 5.4–7.8 minutes for the rapid decline phase and 1.4–1.9 hours for the slow decline phase. In sum, DLX1008 is present, but rapidly cleared from the blood stream of treated mice.

**Fig 2:**
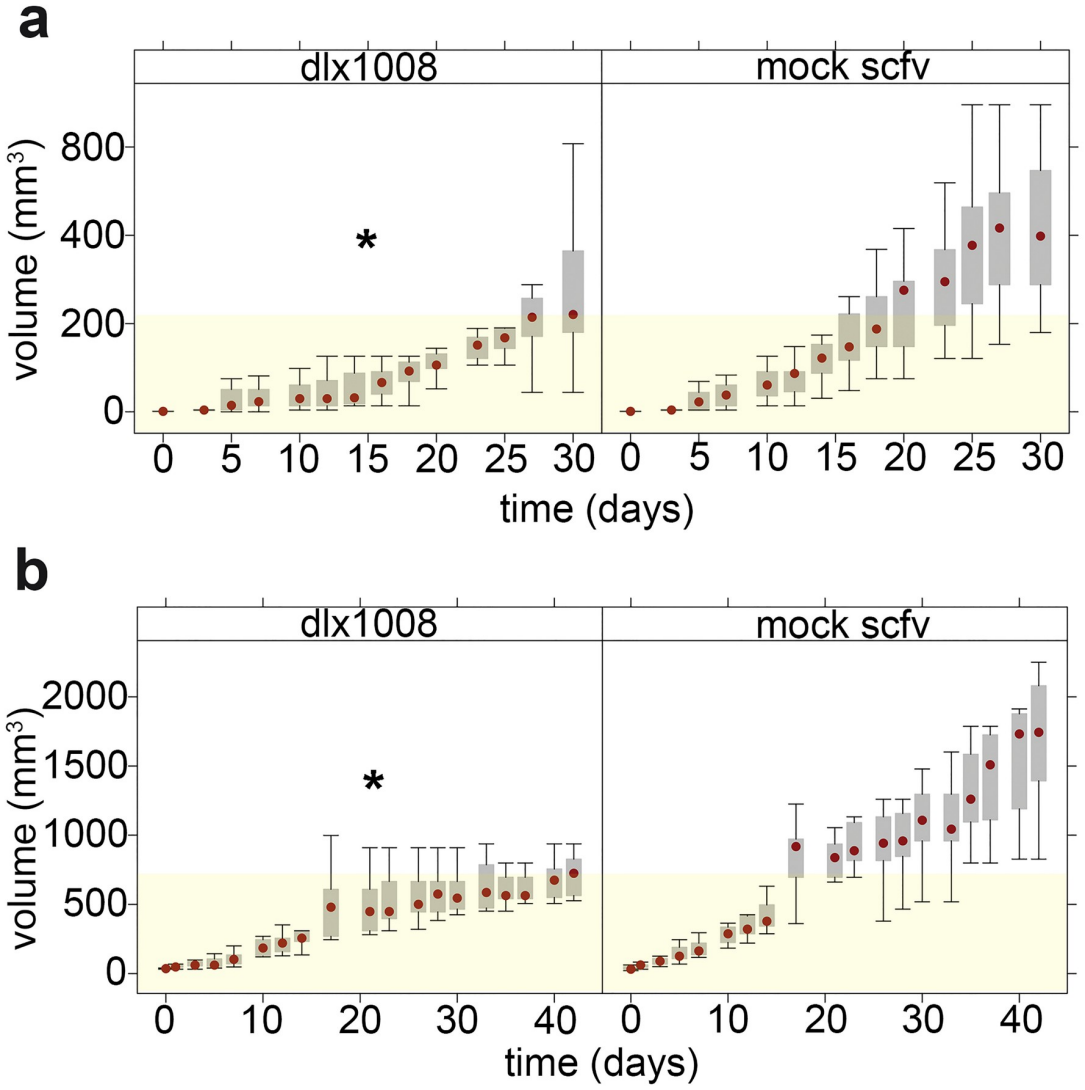
DLX1008 slows the growth of mSLK-KSHV xenograft tumors. Tumor volume, as measured by calipers over the course of treatment in trial 1 (A) and trial 2 (B), is represented by a box and whisker plot comparing the active drug group DLX1008 with the control drug group (mock scFv). Shown are the median (red dot), 25–75% gray box and extreme values (lines). Yellow shading denotes the median tumor volume reached by the DLX1008 group for each trial. Each trial was independently analyzed by random-effect linear model for significance. Both trials independently demonstrated statistically significant increases in tumor volume over time for the nontreatment group compared to the treatment group. * p ≤ 0.05.

We conducted two biologically independent tumor studies. In both, a set of animals were implanted with 5 x 10^5^ cells in Matrigel on the left flank (single tumor per animal) and injected IP for 5 days per week (M/T/W/Th/F) with either 15 mg/kg/day of DLX1008, 15 mg/kg/day of mock scFv, or PBS. In the first experiment (Fig 2A), all animals were treated for 21 days, all animals were euthanized at 30 days after treatment, and tumors were analyzed by histochemistry at endpoint. There were no deaths within the treatment timeline attributable to metastatic spread, and there were also no known toxicities or adverse effects attributable to the drug treatments. In the second experiment (Fig 2B), animal treatment was extended to 41 days, at which point the treatment was stopped. In both trials, tumors were measured regularly with calipers, and the tumor volume was calculated. Tumor volumes were analyzed for each trial independently utilizing a random-effects linear model. (Fig 2) shows a plot of tumor volume for each trial with treatment (DLX1008) and mock scFv-treated groups, with yellow shading to delineate the median volume of the untreated group at termination. Both trials indicated significantly lower tumor growth for the active drug DLX1008 compared to the inactive scFv (p ≤ 0.05 by log-rank test).

The animals in the second experiment were followed until tumors reached 2 cm in any dimension or until animals developed overt morbidity as defined by institutional guidelines. After cessation of treatment, animals declined in health, irrespective of treatment group. This suggests that continuous administration of DLX1008 is needed to arrest growth of the primary tumor xenograft. KM log-rank analysis indicated that there was no difference in survival (p > 0.05) between DLX1008 and combined control groups (PBS and mock scFv).

**Fig 3:**
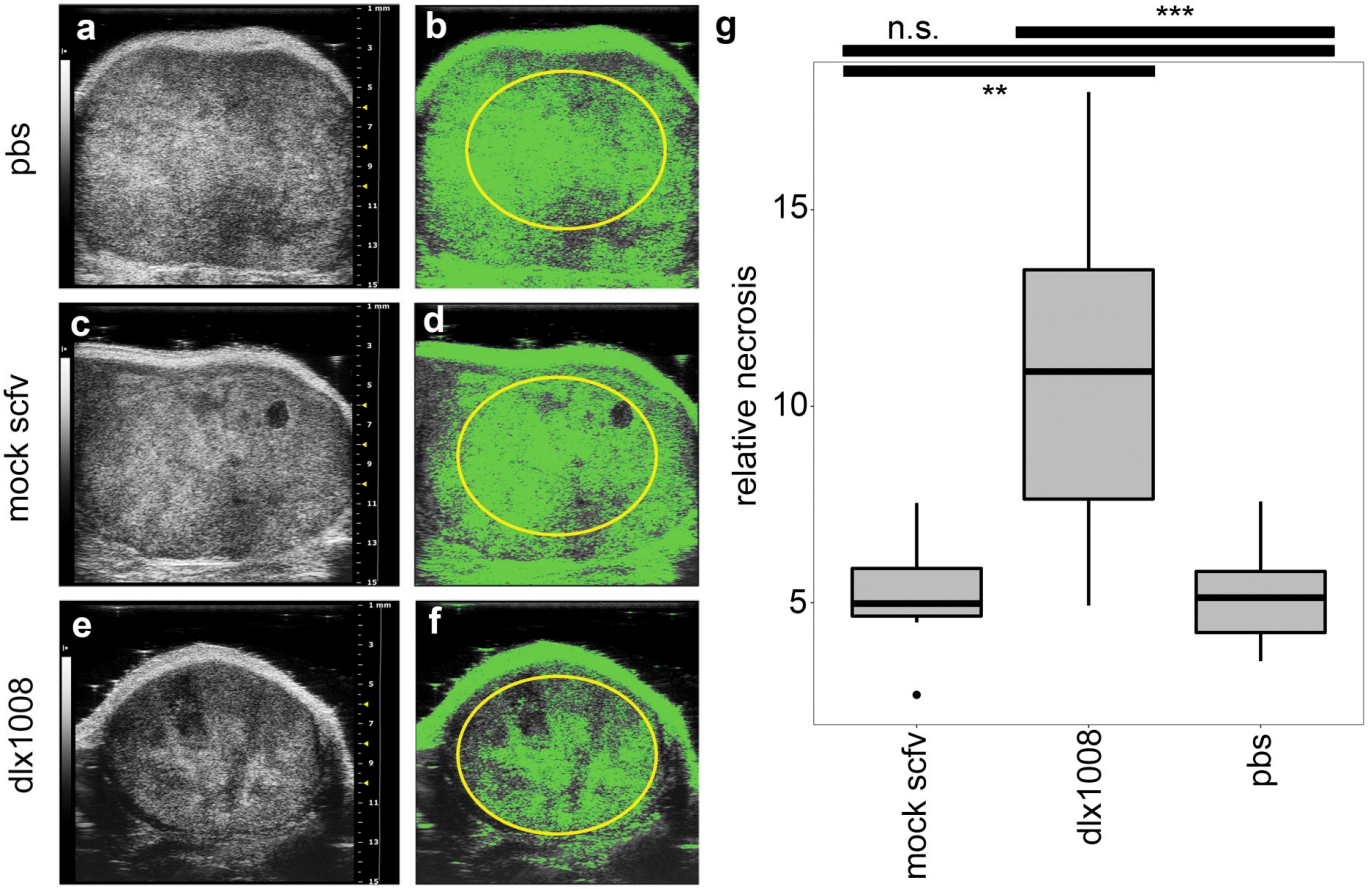
Ultrasound imaging shows decreased density in treatment tumors. B-mode ultrasound images of representative subcutaneous tumors for each group are shown in (A, C, E). Dense, non-necrotic tissue was highlighted in green using ImageJ pixel thresholding, shown in (B, D, F). Necrosis quantification was performed by excluding highlighted pixels from analysis within the selection area (yellow oval). Images are representative of mean quantification score for each group. Percent necrosis was plotted as demonstrated in (G) for all three groups, and significance was determined using multiple comparisons of means with Dunnett contrasts. DLX1008 has significantly more necrosis than mock scFv and PBS, and there was no difference between the control groups. ***p ≤ 0.0001, **p ≤ 0.001, n.s. = not significant.

There are drawbacks to measuring tumor growth by external means only; the composition and density of the tumor are important components to evaluate and not accessible by external visual inspection. To improve upon external tumor measurements as the sole outcome measure, tissue density was measured via ultrasound. This approach tested the hypothesis that even though the tumors were not shrinking macroscopically, viable tumor cells were reduced to necrotic tissue as indicated by a decrease in ultrasound backscatter intensity (hypoechoicity). Using the definition of necrosis as outlined by Majno and Joris [55], we defined it as secondary karyolytic cell death without identification of a specific mechanism. Necrosis was quantified as percentage of a fixed area delineated within the tumor capsule. The size, shape, and scale of the quantified area was consistent between compared groups. ImageJ software was used to determine a consistent threshold and quantify the pixel density of the fixed region; lighter areas are denser/more echogenic, and darker areas are less dense/hypoechoic. Representative images as determined by the median percent necrosis of each group are shown in (Fig 3). Green shading indicates pixel density that is above the threshold for classification as solid tissue (non-necrotic). Pixel density below this threshold was quantified as necrotic and converted to a percentage total area of the fixed region. Comparison of the percentages was done using multiple comparison of means with Dunnett contrasts. DLX1008 caused greater tissue necrosis than PBS treatment alone (p ≤ 0.0001) and isotype control treatment (p ≤ 0.001) when measured by percent area, while there was no difference in the PBS and isotype control groups (p = 0.71). Therefore, we concluded that DLX1008 was responsible for a decrease in central tumor density compared to controls. Thus, the slower-growing DLX1008 tumors were also of lower-density than their mock-treated counterparts, suggesting that caliper measurements of external volume underestimated the treatment’s effect and that the tumors were disintegrating from the inside out, consistent with reduced access to oxygen and nutrients.

To independently probe the integrity of the experimental tumors, integrin αVβ3 expression was measured in trial 2 by transdermal fluorescent expression after IV injection of the NIR-responsive IntegriSense 750 reagent (PerkinElmer, Inc). Integrin αVβ3 is highly expressed in KS and our KS tumor model [56]. In fact, integrin αVβ3 is considered a receptor for KSHV [57]. The expectation would be (a) that tumors with reduced cellularity and/or reduced angiogenesis would take up less probe and (b) that dead cells would express less integrin αVβ3. First, we evaluated if the integrin was expressed in a mSLK-KSHV xenograft (S2 Fig). A representative athymic nude mouse is shown from different angles at various timepoints. The mouse is shown in anterior and lateral view at 4h, where high nonspecific signal is present, and at 24h, where nonbinding probe has cleared the system and signal remains at integrin-rich sites. Arrowheads indicate the site of the subcutaneous tumor, which retains specific signal.

**Fig 4:**
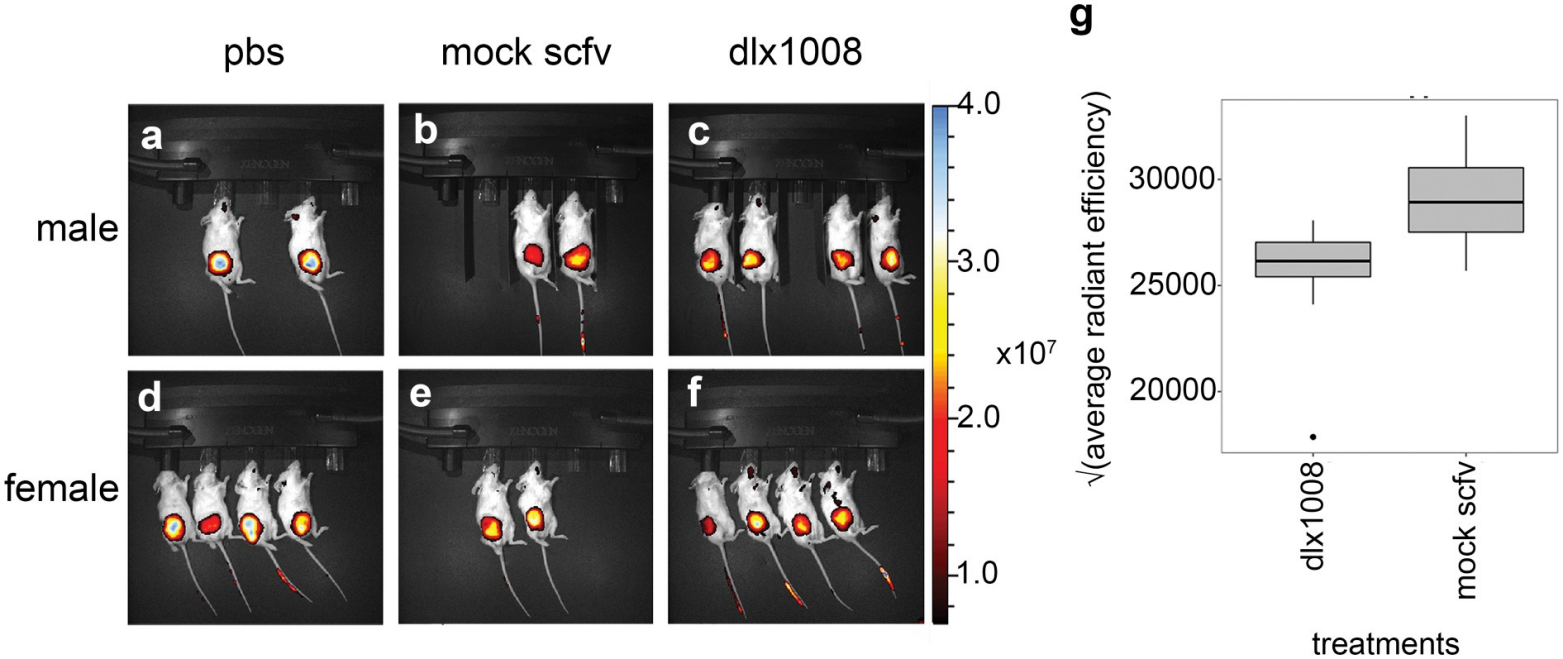
Integrin expression decreased in treated tumors. Animals at the end of the study were imaged before and 24h after IV injection of a near-infrared fluorescent integrin probe. Mice at 24h are shown in (A-F) as white light images with integrin expression as a pseudo color overlay. Mice were imaged in groups male (A-C) and female (D-F), and the treatment groups PBS (A, D), mock (B, E), or DLX1008 (C, F). Automatically generated ROIs determined the change in average radiant efficiency from background measurements as a fluorescence measurement, and the square root of the values were plotted in (G) for DLX1008 and mock scFv. DLX1008 was calculated to statistically express significantly lower levels of integrin compared to isotype control scFv (p ≤ 0.05). There was no difference in integrin expression between male and female groups.

Next, treated and control NSG mice were measured. The experiment was conducted at day 41 of treatment; hence, fewer animals were available for PBS and control groups than for DLX1008 treated groups. Measurements were conducted at baseline (0h) and then 24 hours after injection of the integrin probe (24h). The instrument settings were standardized before imaging with all mice at both 0h and 24h, and the average radiant efficiency was determined for each mouse by using the automatically-generated region-of-interest (ROI) at 24h and subtracting the same ROI from the 0h data on file for the corresponding animal. A square root transformation was utilized for average radiant efficiency to account for large values and normalize data for display. (Fig 4) shows the mice at 24h, with fluorescence indicating the integrin-bound probe expressed as a color overlay. There was a significant difference (p ≤ 0.05) in the treatment (DLX1008) and mock (scFv isotype control) groups, indicating decreased average integrin expression in treated tumors. There was no significant difference between male and female groups, or between PBS and scFv isotype control groups. Combined with previous fluorescent Arg-Gly-Asp (RGD) -imaging data confirming that our xenograft model overexpresses integrin αVβ3 (S2 Fig), this suggested that DLX1008 caused a decrease in integrin-positive tumor mass consistent with a loss of density.

**Fig 5:**
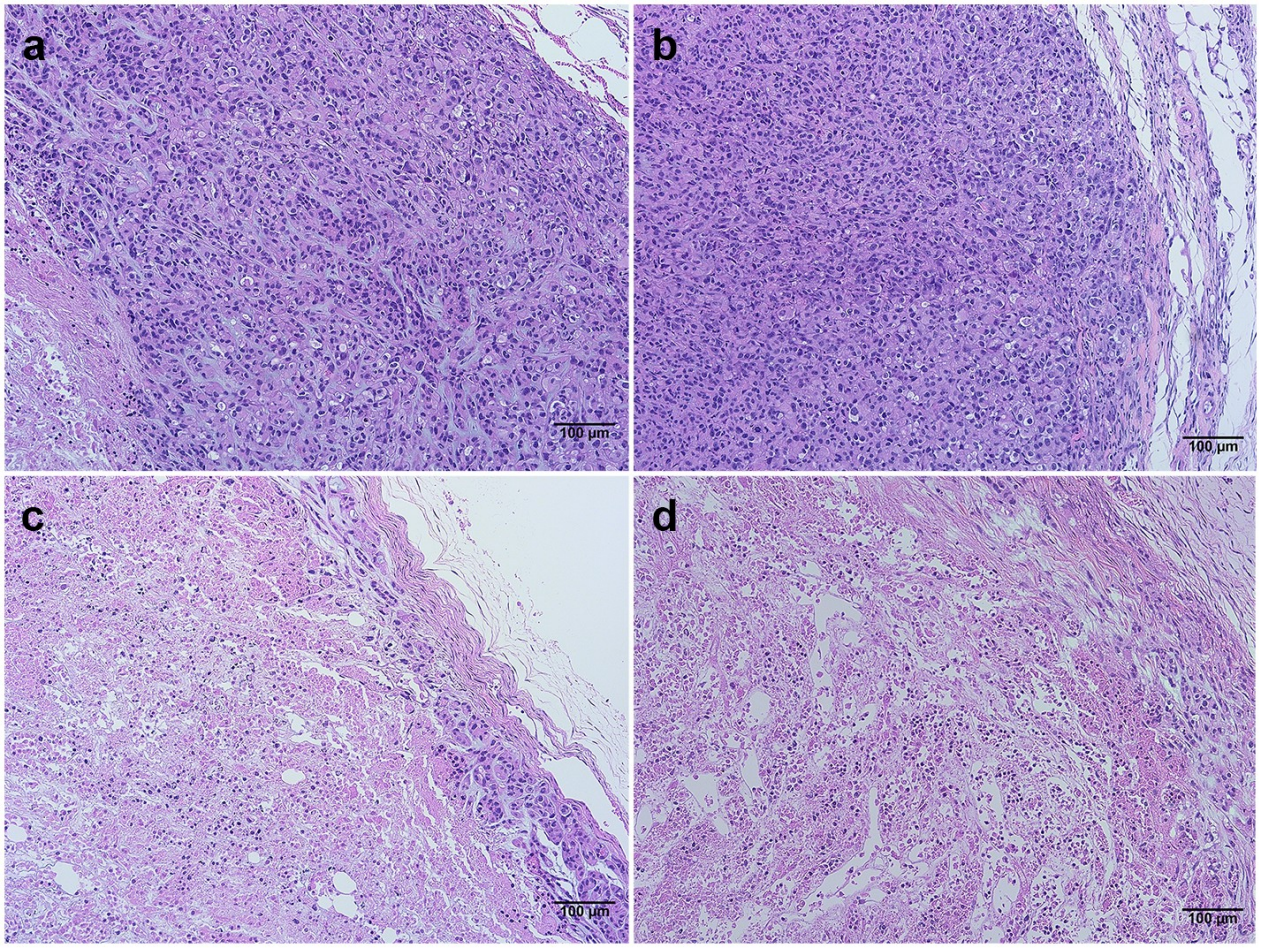
Hematoxylin and eosin stains of tumor tissue show morphological differences. H&E stains are of tumor cross-sections. Images are representative of mock scFv control group (A, B) and DLX1008 treatment group (C, D). All scale bars are 100 μm.

Finally, we evaluated internal tumor structure by histology. Tumor tissue (both trials) and kidney tissue (trial 1) from the mice were formalin-fixed, embedded, sectioned, and stained for hematoxylin and eosin (H&E). Kidney tissue collected in the initial trial showed no signs of abnormality or damage in either the biologically active or inactive drug groups compared to PBS-treated mice. Tumor sections were examined and compared using light microscopy at low magnification to discern morphological differences. Despite being smaller volumetrically, treated tumors (DLX1008) had qualitatively significant central necrosis and marginal hypoxia which extended closely to the outer tumor capsule (Fig 5). Histology of formalin-fixed, paraffin embedded tumor tissue was examined by staining tissue sections with hematoxylin and eosin (H&E). Images were captured that are representative of mock scFv control group (Fig 5A and 5B) and DLX1008 treatment group (Fig 5C and 5D). The outer edge of the tumor is included in each image for reference. Control-treated tissues display a thick layer of intact, well-adhered cells from the tumor capsule inward. DLX1008-treated tissues have sparse intact cells near the capsule that separate and are not well-adhered, and below the surface is cell debris, RBCs and inflammatory infiltrate. The DLX1008 group also had central necrosis that regularly extended to the tumor edges, indicative that there was hypoxia sufficient to cause tumor cell death. Control group tumors, in contrast, displayed robust margins of healthy tumor cells. This correlated with the noninvasive imaging data to further elucidate the hypoxic effect of DLX1008 on KS-like xenograft tumors.

## Discussion

These experiments demonstrate that DLX1008 is an effective angiogenesis inhibitor and has anti-tumor activity against KS in a small animal model. To improve on superficial visual measurements of tumor growth, we utilized several imaging techniques to determine that treated tumors (a) were of decreased echogenic density, (b) had decreased expression of tumor-cell-associated integrin or less vasculature for the probe to reach the tumor, and (c) histologically indicated a more extensive degree of central, deep necrosis. DLX1008 also reduces tumor growth in a preclinical model of glioblastoma [23]. This establishes DLX1008 as an effective agent to reduce tumor vasculature and thereby tumor growth.

Although DLX1008 resulted in significant, easily visible tumor inhibition, simple caliper-based size measurements underestimated the efficacy of this and presumably other angiogenesis inhibitors in this xenograft model. The same rationale would apply to other studies that evaluate angiogenesis inhibitors in subcutaneous tumor graft models, including PDX models, by caliper-based size measurements alone. Repeated, non-invasive ultrasound imaging was validated as an economical, reliable and robust method to evaluate blood circulation and tumor health. Computerized image analysis can be applied to grayscale B-mode ultrasonography images to acquire quantitative data (reviewed in [58]); ImageJ is one such program that has been utilized to obtain reliable data in human muscle tissue [59, 60]. The performance of this type of image analysis was validated here.

The KS-mimic tumor xenografts died from the inside-out in response to DLX1008 treatment, exclusively affecting tumor cells and leaving surrounding mouse tissue intact. Detailed histologic evaluation showed that the necrotic areas were filled in with extracellular matrix, and an outer layer of healthy tumor cells maintained much of the overall shape and size, despite the tumor mass being mostly acellular. The primary determinant of differences in echogenicity between tissues undergoing different types of cell death is the chromatin/nuclear structure. Cells undergoing mitotic arrest and apoptosis display increased echogenicity due to condensation of chromatin, whereas oncotic or destructive cell death causes a decrease in echogenicity [61, 62]. This is an important consideration when evaluating echogenicity of tissues as a measure of response. Cytotoxic therapies which directly arrest mitosis may have the opposite appearance on B-mode ultrasonography compared to therapies that affect angiogenesis and result in hypoxic cell death.

Essential aspects of the angiogenic phenotype of KS are recapitulated in KSHV-driven EC culture models. Infection with KSHV increases the permeability of EC via phosphorylation of VE-cadherin and β-catenin, leading to internalization and disruption of EC junctions [37, 38]. Infection of lymphatic EC (LEC) with KSHV upregulates membrane type-1 matrix metalloproteinase (MT1-MMP), which causes vascular basement membrane degradation [35, 63]. KS transcription signatures show the greatest similarity to LEC, though they do not faithfully represent either LECs or blood ECs (BECs); rather, KSHV can infect either cell type and drive gene expression toward that of the other [4, 36, 64]. Endothelial-to-mesenchymal transition (EMT) is associated with KSHV infections of LEC [35]; however, there also exists evidence for KSHV-induced mesenchymal-to-endothelial transition (MET) [15, 40, 65, 66]. Our understanding of the underlying mechanisms of this reprogramming is far from complete, though emerging evidence has implicated the viral microRNAs [67–69]. The ability of KSHV to induce differentiation or dedifferentiation depending on the cell of origin is consistent with the known multifocal and heterogeneous presentation of KS *in vivo*. Enhanced, disorganized angiogenesis and EC dysfunction define the pathology of reactive KS lesions. These characteristics are difficult to recapitulate in tissue culture; hence, small animals are irreplaceable for the study of treatment modalities that target angiogenesis and the tumor microenvironment.

Anti-VEGF antibodies operate in the tumor microenvironment, whereas VEGF receptor tyrosine kinase inhibitors (RTKI) act within the tumor cell (reviewed in [70]). This leads to different mechanisms of resistance development. Tumor cells rapidly develop resistance to RTKI by selection of mutations of the active site or in “gate-keeper” residues that define drug specificity. Drug resistance is irreversible, prompting the need for second, third, and fourth-line RTKI regimens, each associated with increasing toxicity [71]. By contrast, the tumor response to indirect angiogenesis inhibitors is adaptive and reversible. The untransformed EC which drive angiogenesis are not hypermutable, are periodically replaced, and typically do not develop resistance to VEGF inhibitors.

The advantages and disadvantages of anti-angiogenic versus cytotoxic cancer therapy have been the subject of extensive debates in solid tumor oncology. On one hand, anti-angiogenesis regimens are not curative [19]; they are tumor-static rather than tumor-toxic. On the other hand, cytotoxic therapy has reduced efficacy against slow-growing, “smoldering” tumors; here, anti-angiogenic drugs have shown efficacy [72]. This led to the current practice of bevacizumab-adjuvant therapy in a range of human cancers, including KS [18], rather than relying on anti-angiogenesis inhibitors as single agent. This treatment paradigm was recapitulated in this mode. Treatment with DLX1008 alone showed activity but was not curative. DLX1008/ brolucizumab, like bevacizumab, would have to be combined with other active agents to induce curative tumor regression.

Our thinking about the mechanism of action for adjuvant anti-angiogenic therapy is rapidly evolving. Initially, VEGF-targeting therapies were thought to function additively to cytotoxic chemotherapy by reducing nutrient flow directly. More recently, their chemo-sensitizing power has been recognized as well. As the tumor vasculature normalizes secondary to vessel pruning by VEGF inhibitors, drug access to the tumor interior is increased [27, 63, 70, 73, 74]. There is evidence that vascular normalization can be biphasic and inversely drug concentration-dependent, as measured by tumor blood flow [73]. Optimal therapies would normalize vasculature enough to access the tissue with cytotoxic compounds, but not be so prolonged as to completely degrade vascular access and force hypoxia-induced-tumor evolution to VEGF independence [63]. Tumor cells respond to hypoxic stress induced by angiogenesis inhibitors with metabolic switching and the secretion of different cytokines. Hypoxia induction is of particular importance in KS, since KSHV responds to hypoxia with reactivation [45, 75–77]. Many hypoxia-inducible genes of KSHV are pro-tumorigenic and will drive cell growth. In the special case of KS, hypoxia also induces new targets for combination therapy, such as the viral ORF36 and ORF21 kinases, which convert ganciclovir and AZT into cytotoxic metabolites [78], thus opening up the possibility of combination therapy between an angiogenesis inhibitor and an anti-viral agent.

## Supporting information

S1 Fig Cell line mSLK-KSHV maintains characteristics of KS tumors and endothelial cells. Shown is a karyogram representation of the 22 human autosomes based on CGH analysis for the following samples (left to right): HUVEC_KSHV clone 1, HUVEC_KSHV clone 2, HUVEC_KSHV clone 3, HUVEC stock DD, HUVEC stock BD, TIVE-E1, TIVE-E1mu clone A, TIVE-E1mu clone B, TIVE-L1, TIVE-L1mu clone A, TIVE-L1mu clone B. Uninfected, immortalized HUVEC cultures are overlaid in yellow. Amplifications were in red, copy number losses in blue. These are scaled and normalized to ±2 copies. Gray bars underneath indicate the HUVEC group, and dark green bars indicate the TIVE group. Stars indicate chromosomes which share a CGH pattern with Caki-1 cells. Chromosome 2 represents an example for the genomic diversity among different clones. All HUVEC-derived cells are normal, except for focal amplification on the tip of 2p in one sub clone. All TIVE-derived cells share large allelic loss and focal amplification in 2q with Caki-1. Only the two tissue culture isolates show localized amplifications in the telomeric regions of 2q and 2 p, the four mouse-tumor derived isolates do not. A similar pattern is evident for chr. 17, whereas the chromosome abnormalities in chr. 19 and 12 differ for each cell line.
(DOCX)

S2 Fig Integrin is overexpressed in mSLK-KSHV tumors. Nude mice were implanted with a xenograft mSLK-KSHV subcutaneous tumor on the flank and injected IV with an RGD near infrared (NIR) probe. Fluorescent signal at 800nm was measured on the Pearl Trilogy (Li-Cor) at 4h and 24h post-injection. Pseudo color fluorescent intensity is overlaid on the white light photo (first image). White arrowheads indicate the tumor site.
(DOCX)

S1 Checklist The ARRIVE guidelines checklist. (DOCX)
