# Supplementary figures and images for: DLX1008 (brolucizumab), a single-chain anti-VEGF-A antibody fragment with low picomolar affinity, leads to tumor involution in an in vivo model of Kaposi Sarcoma
Source: PLoS One. 2020 May 14;15(5):e0233116. doi: 10.1371/journal.pone.0233116 (PMC7224538; doi:10.1371/journal.pone.0233116)

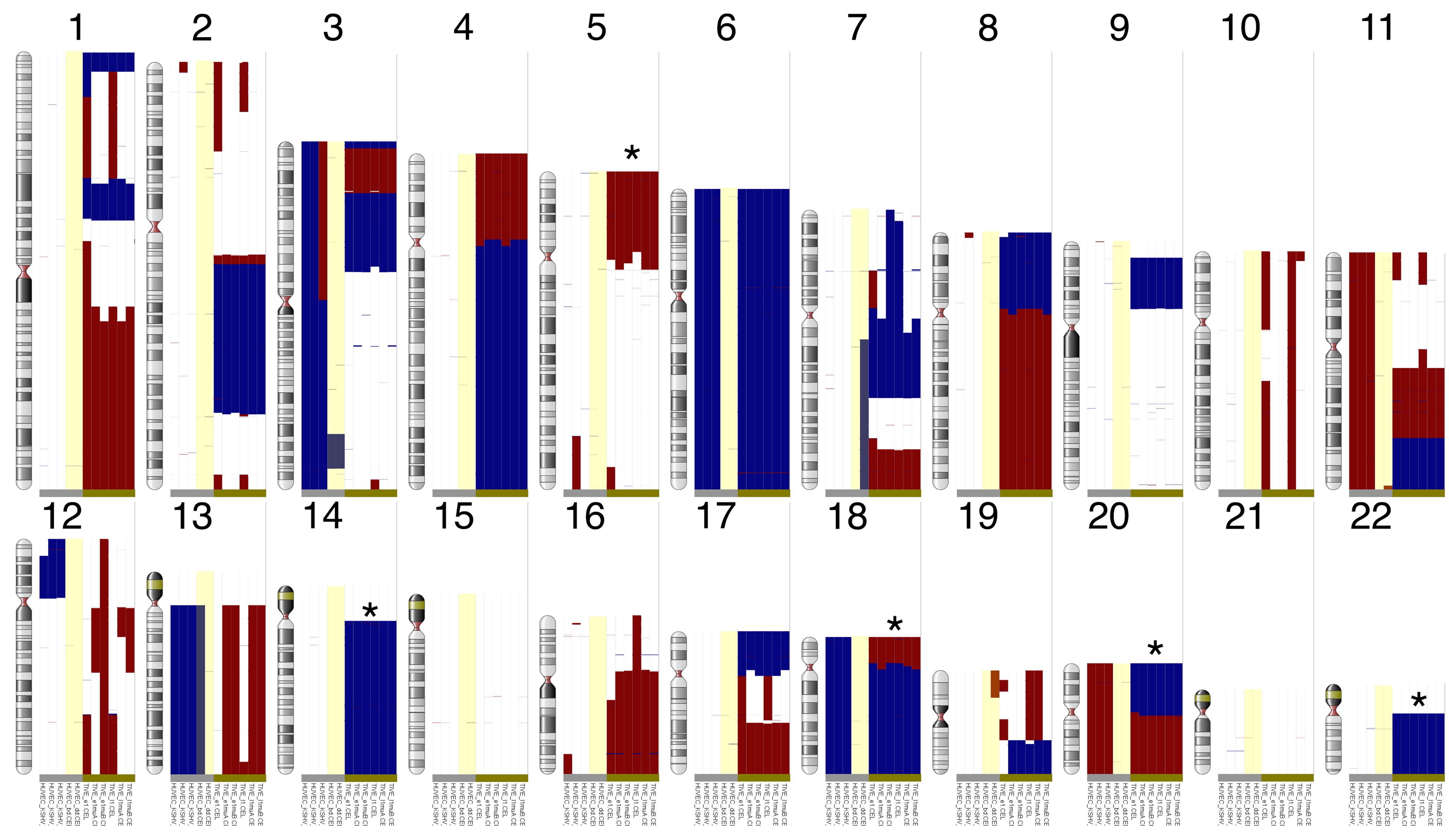

Supplement: S1 Fig — Shown is a karyogram representation of the 22 human autosomes based on CGH analysis for the following samples (left to right): HUVEC_KSHV clone 1, HUVEC_KSHV clone 2, HUVEC_KSHV clone 3, HUVEC stock DD, HUVEC stock BD, TIVE-E1, TIVE-E1mu clone A, TIVE-E1mu clone B, TIVE-L1, TIVE-L1mu clone A, TIVE-L1mu clone B. Uninfected, immortalized HUVEC cultures are overlaid in yellow. Amplifications were in red, copy number losses in blue. These are scaled and normalized to ±2 copies. Gray bars underneath indicate the HUVEC group, and dark green bars indicate the TIVE group. Stars indicate chromosomes which share a CGH pattern with Caki-1 cells. Chromosome 2 represents an example for the genomic diversity among different clones. All HUVEC-derived cells are normal, except for focal amplification on the tip of 2p in one sub clone. All TIVE-derived cells share large allelic loss and focal amplification in 2q with Caki-1. Only the two tissue culture isolates show localized amplifications in the telomeric regions of 2q and 2 p, the four mouse-tumor derived isolates do not. A similar pattern is evident for chr. 17, whereas the chromosome abnormalities in chr. 19 and 12 differ for each cell line. (DOCX) [file pone.0233116.s001.docx]

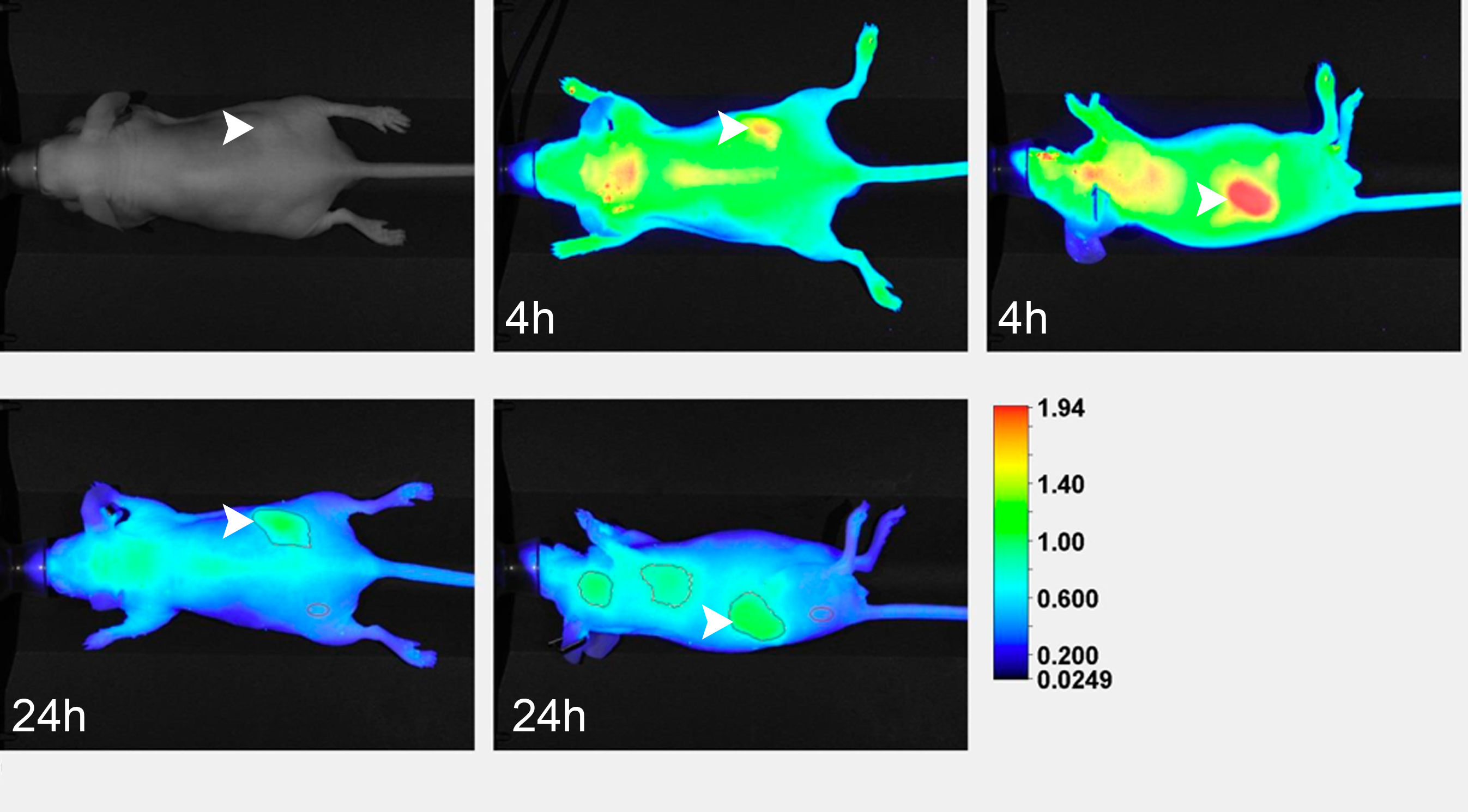

Supplement: S2 Fig — Nude mice were implanted with a xenograft mSLK-KSHV subcutaneous tumor on the flank and injected IV with an RGD near infrared (NIR) probe. Fluorescent signal at 800nm was measured on the Pearl Trilogy (Li-Cor) at 4h and 24h post-injection. Pseudo color fluorescent intensity is overlaid on the white light photo (first image). White arrowheads indicate the tumor site. (DOCX) [file pone.0233116.s002.docx]
